# Autophagy and Mitochondrial Homeostasis During Infection: A Double-Edged Sword

**DOI:** 10.3389/fcell.2021.738932

**Published:** 2021-09-03

**Authors:** Sutian Wang, Kunli Zhang, Yuchang Yao, Jianhao Li

**Affiliations:** ^1^State Key Laboratory of Livestock and Poultry Breeding, Guangdong Key Laboratory of Animal Breeding and Nutrition, Institute of Animal Science, Guangdong Academy of Agricultural Sciences, Guangzhou, China; ^2^Institute of Animal Health, Guangdong Academy of Agricultural Sciences, Guangdong Provincial Key Laboratory of Livestock Disease Prevention Guangdong Province, Guangzhou, China; ^3^College of Animal Science and Technology, Northeast Agricultural University, Harbin, China; ^4^Maoming Branch, Guangdong Laboratory for Lingnan Modern Agriculture, Maoming, China

**Keywords:** autophagy, homeostasis, dual role, pathogen infection, mitochondria

## Abstract

Autophagy, an essential biological process that affects immunity, is a powerful tool that host cells can use to defend against infections caused by pathogenic microorganisms. Autophagy can not only initiate innate immune responses but also degrade the cellular components that provide the conditions for removing the invaders. However, hyperactivated or inhibited autophagy leads to mitochondrial dysfunction, which is harmful to the host itself and is involved in many types of diseases. Mitochondria perform the functions of biological oxidation and energy exchange. In addition, mitochondrial functions are closely related to cell death, oxygen radical formation, and disease. Accumulation of mitochondrial metabolites affects survival of intracellular pathogens. In this mini-review, we focus on the crosstalk between autophagy and mitochondrial homeostasis during infection.

## Introduction

Pathogenic microorganisms are widely distributed in nature. After they invade into animals, these microorganisms start to grow and release toxins or toxic metabolites and cause damage to the host. A typical characteristic of the diseases caused by pathogenic microorganisms is infectious. That is, when these diseases occur, they always spread in the crowds and have a serious impact on public health and the animal breeding industry. For a long time, autophagy was thought to be induced by starvation (Mizushima et al., 1998). Activation of autophagy was known to help degrade damaged organelles and harmful metabolites (Qi and Chen, 2019). However, research has shown that autophagy not only exists in healthy bodies, but also found in diseased bodies (Levine and Kroemer, 2008). Autophagy is generally recognized as a cellular clearance system, where it clears pathogens through the autolysosome (Inomata et al., 2020). Furthermore, autophagy also activates host adaptive immunity by regulating antigen presentation and maintaining physiological homeostasis (Valecka et al., 2018). However, there is also evidence indicating that autophagy can aggravate pathogenic infection and induce physiology disorders (Wang et al., 2019). Moreover, hyperactivated or inhibited autophagy leads to mitochondrial dysfunction, which is harmful to host itself (Shintani and Klionsky, 2004). Mitochondria are the main sites where biological oxidation and energy conversion occur and are closely associated with many diseases. Many studies have shown that the mitochondrion plays an important role in regulating various cellular physiological activities and immune responses upon infections (Tiku et al., 2020). Since mitochondrial function affects the invasion, clearance, and immune escape of several pathogens, maintaining mitochondrial homeostasis is an effective strategy to maintain host health.

Bacterial and viral pathogen-associated molecular patterns (PAMPs) can trigger autophagy and induce professional immune cells to produce mitochondrial metabolites, including reactive oxygen species (ROS) and reactive nitrogen species (RNS) (Wang et al., 2020). Moderate ROS and RNS levels help to eliminate pathogens; however, excessive amounts of ROS and RNS disrupt mitochondrial homeostasis and further damage to the tissues and organs of the host (Shadel and Horvath, 2015). The relationship between autophagy and infection has long puzzled researchers. Is autophagy the host’s own defense system that fights against the invasion of pathogens or a collaborator that helps pathogens to achieve invasion or immune escape? Is autophagy a trigger for cell death or a simultaneous cellular response that accompanies cell death? Does autophagy help the host defend against disease or is it a physiological disorder induced by disease? Since the role of autophagy varies depending on the pathogen and the infected individuals, these questions are difficult to answer definitively. Here, we discuss the dual role of autophagy from the perspective of mitochondrial homeostasis, and we also summarize the crosstalk between autophagy and the mitochondrion.

## Dual-Role of Autophagy During Infection

Autophagy degrades intracellular components or organelles to maintain physiological cell homeostasis. It is generally known that autophagy is involved in many diseases, such as cancer, metabolic disorders, neurodegeneration, and infection. Vps34-Beclin1 complex is essential for formation of autophagic vacuole. A single allele deletion mutation of Beclin1 is present in all kinds of cancer including breast cancer, prostate cancer, lung cancer, and liver cancer (Qu et al., 2003). Overexpression of Beclin1 helps inhibit growth of thymic cancer cell (Liang et al., 1999). However, autophagy also protects cancer cell from apoptosis or necrocytosis (Degenhardt et al., 2006). Inhibition of expression of ATG5 and ATG7 promotes anoikis of cancer cell via suppression of autophagy (Fung et al., 2008). Autophagy is also a critical mediator of pathological response during diabetes procession (Yamamoto et al., 2018). Beclin1-mutated mice became more sensitive to insulin and reduced risk of diabetes (Kuramoto et al., 2021). Activation of Beclin1 in fat cells makes diabetics sensitive to insulin in turn (He et al., 2013). The mechanism is that the mutation in Beclin1 leads to separation of the Bcl2-Beclin1 complex. And then the free Beclin1 interacts with exocyst proteins in white adipose tissue to promote adiponectin secretion into the blood that improves insulin sensitivity. Moreover, autophagy helps to isolate pathogenic microbes in a closed environment and eliminate them by intracellular acidification and a variety of enzymes (Weiss and Schaible, 2015). Pathogens typically invade cells via endocytosis, and are then transported to the lysosome for degradation. However, several pathogens have evolved ways to escape from the immune response. Some pathogens can inhibit autophagy by preventing formation of autolysosome or by directly hijacking and utilizing the autophagosome for their own survival and proliferation. The following highlights the dual-role of autophagy in infection prevention.

### Autophagy Not Only Facilitates the Clearance of Pathogens, but Also Gets Manipulated by Pathogens

Once a pathogen invades the cytoplasm of the host, it is typically encased in an autophagosome, which degrades the pathogen after fusion with the lysosome. However, the process of autophagy leading to clearance of pathogens is different depending on the pathogen. Rapamycin, an inducer of autophagy, promotes the clearance of *Mycobacterium tuberculosis* by macrophages (Deretic et al., 2006). When *Streptococcus* is taken into cells, NRLP4 is recruited to bacterial autophagosome-like vacuoles, which binds to ARHGDIA to regulate xenophagy (Nozawa et al., 2017). *Salmonella* forms Salmonella-containing vacuoles (SCVs) inside cells. In macrophages, some mycoproteins produced by *Salmonella* induce mitochondrial damage and trigger mitophagy (Manzanillo et al., 2013). The *Salmonella* escaped from its type III secretion systems can be cleaned by lysosome (Birmingham et al., 2006), but in epithelial cells, free *Salmonella* are ubiquitinylated, colocalize with LC3 and p62, and are eventually encapsulated in the autophagosome and degraded (Perrin et al., 2004). Autophagy-associated proteins ATG5, Beclin-1, and p62 all participate in the elimination of the Sindbis virus (Liang et al., 1998; Orvedahl et al., 2010; Sumpter and Levine, 2011). SHISA5-mediated autophagy inhibits the replication of the hepatitis C virus (Kim et al., 2016). In addition, autophagy can load the pathogen antigens onto the MHC-II complex to trigger T-cells, thereby initiating a specific immune response. For example, rapamycin can improve the efficiency of *M. tuberculosis* antigen presentation by antigen-presenting cells (Jagannath et al., 2009). Autophagy is also involved in presenting EBV and HIV-1 antigens (Paludan et al., 2005; Kyei et al., 2009).

However, autophagy does not all promote the clearance of all pathogens. In some cases, autophagy promotes the survival and proliferation of pathogens. These pathogens inhibit, destroy, and even manipulate autophagy in multiple ways. *Salmonella Typhimurium* can inhibit the initiation of autophagy by regulating mTOR activity (Tattoli et al., 2012). The γ134.5 protein of HSV-1 prevents formation of the autophagosome by competitively binding to Beclin-1, and HSV-1 also inhibits autophagy via the downregulation of p62 and OPTN (Waisner and Kalamvoki, 2019). Some pathogens inhibit autophagy by influencing the activity of autophagy components. The T4SS effector RavZ of *L. pneumophila* can bind to the C-terminus of LC3 to obstruct autophagosome formation (Choy et al., 2012). The viral proteins VP48, VP122, and VP132 of the Singapore grouper iridovirus can competitively bind to ATG5 and restrain LC3 conversion (Li et al., 2020). *Staphylococcus aureus* can activate autophagy through inhibiting the cellular cAMP-EPAC-RAP2B pathway. Subsequently, these bacteria hide in autophagosomes and continue to proliferate, while inhibiting the fusion of the autophagosome and lysosome (Schnaith et al., 2007; Mestre and Colombo, 2012). Viral proteins 2BC and 3A of poliovirus cause accumulation of autophagosomes in the host cells, and its replication depends on these autophagosomal structures (Jackson et al., 2005). Thus, autophagy has multiple physiological and pathological functions, including elimination of pathogens and activation of the innate immune response of the host. However, some pathogens have evolved methods to evade autophagic degradation.

Therefore, it is important to know how to regulate autophagy to play a positive role in the process of battling against pathogenic microorganism. The invasive mycobacterium tuberculosis can hide in endosomes and continue to reproduce. Rapamycin-induced autophagy can combine with bacteria-containing endosomes to form autophagolysosomes, which degrade intracellular bacteria (Deretic et al., 2006). The survival of *P. gingivalis* around the gums depends on autophagy. After invading the host cells, *P. gingivalis* are encapsulated in autophagosomes and reproduce in them. Autophagy inhibitor (3-methyladenine or wortmannin) treatment causes internalized *P. gingivalis* transits to the phagolysosome where it is destroyed and degraded (Belanger et al., 2006). Replication of poliovirus, rhinovirus, and mouse hepatitis virus depends on autophagy-like structure. 3-methyladenine treatment decreases these viruses yield (Jackson et al., 2005). It can be known from the above that the proliferation of pathogenic microorganisms can be suppressed by activation or inhibition of autophagy in the process of different pathogenic infection according to the certain conditions.

## Normal Mitochondrial Function Affects Infection

As noted above, autophagy has dual roles during pathogens infection. Normal mitochondrial function in infected cells is an important factor influencing the function of autophagy. All life on earth requires energy, and in animal cells, energy conversion depends on mitochondria. Mitochondria, organelles found in most eukaryotic cells, except mature mammalian erythrocytes, generate over 80% of the energy needed by the cell. In addition, mitochondria are closely related to cell death, oxygen radical formation, and disease. Moreover, some diseases are often accompanied by abnormal mitochondrial function, whereas mitochondrial dysfunction caused by abnormal mitochondrial structure or mitochondrial DNA (mtDNA) mutations is also a key factor of some diseases. Mitochondrial function is also intimately associated with the survival of some pathogens and the ability of some pathogens to evade the host immune system.

When pathogens infect mammalian cells, PAMPs are recognized by pattern recognition receptors (PRRs) on host immune cells and activate innate immune responses. Mitochondria are also involved in this process. Toll-like receptors (TLRs) recognize various microbial components, such as lipopolysaccharide, lipoteichoic acid, peptidoglycan, and teichoic acid mannose. Activation of these TLRs leads to increased mitochondrial ROS levels through the recruitment of mitochondria to the phagolysosome, which triggers NOX (West et al., 2011). Several studies have shown that clearance of pathogens involves ROS. A S. *aureus* mutant with disrupted expression of an antioxidant gene was demonstrated to be more susceptible to oxidative killing of the host (Liu et al., 2005). The OxyR regulon is an important bacterial antioxidant defense system (Pedre et al., 2018). A oxyRS mutant strain of E. coli was shown to be more susceptible than the wild-type strain to human neutrophil-mediated clearing, and further research showed that this oxyRS mutant strain of *E. coli* survives longer in oxidase-deficient neutrophils (Staudinger et al., 2002). It is well accepted that ROS can induce the release of nitric oxide (NO) through activation of iNOS via the NF-κB signaling pathway. NO subsequently induces production of peroxidase and superoxide, which helps to clear pathogens (Heo et al., 2008). In addition, the oxidative damage caused by ROS can directly expose intracellular pathogens to an oxidative environment (Pratico, 2001). Unprotected pathogens exposed to this environment are rapidly degraded by immune cells. In summary, mitochondrial metabolites help remove pathogenic microorganisms.

Besides providing energy for the cell, mitochondria are also involved in cell death; that is, imbalance of mitochondrial homeostasis often leads to cell death. B cell lymphoma 2 (Bcl-2) protein family is the most important family of proteins involved in apoptosis. After the activation of apoptosis, outer mitochondrial membrane permeabilization (MOMP) is induced (Kale et al., 2018). Subsequently, several pro-apoptotic factors, such as cytochrome c and caspases, are then released into the cytoplasm. The apoptosome, comprised cytochrome c and cytosolic protein apoptotic protease activating factor 1, can activate caspase-1 and caspases 3/6/7 consecutively, which eventually leads to apoptosis (Kale et al., 2018). Another class of PRRs closely related to mitochondrial function is the Nod-like receptors (NLRs), which are located in the cytoplasm. Previous evidence has shown that activated NLRP3 inflammasome and its adaptor protein ASC are located within mitochondria (Zhou et al., 2011). Damaged mitochondria release a large amount of mtDNA, cardiolipin, and lipids into the cytoplasm where NLRP3 and NLRC4 inflammasomes can be activated (Iyer et al., 2013; Jabir et al., 2015). These inflammasomes further activate caspase-1, which ultimately leads to inflammatory cell death (Broz and Dixit, 2016). It is difficult to judge if cell death helps to eliminate pathogens or spread them. Cell death plays different roles in cells infected with different pathogens. The type of pathogen is the most critical factor that can cause diametrically opposing results regarding cell death. For example, cell death helps to remove flavivirus, whereas host cell death facilitates dissemination of *Salmonella* (Wemyss and Pearson, 2019; Pan et al., 2021). Thus, some pathogens utilize cell death to obtain nutrients or aid in their dissemination. However, cell death also results in the elimination of the intracellular niche for certain pathogens, further preventing the proliferation of pathogens. Moreover, dead cells can induce moderate innate immune response to the infection.

## Crosstalk Between Autophagy and Mitochondrial Homeostasis

The maintenance of mitochondrial homeostasis depends on the proper folding, assembly, and translocation of mitochondrial proteins. Moreover, mitochondria provide energy to support cellular functions and intracellular environmental changes. During these processes, a large number of metabolic by-products accumulate within mitochondria, including ROS, lipids, and organic acid. The inadequate removal of these by-products disrupts mitochondrial homeostasis, which aggravates infection and causes tissue and organ damage. Autophagy is a key factor that affects mitochondrial homeostasis through controlling the degradation of mitochondrial metabolic by-products and damaged mitochondria (Cho et al., 2020). *Streptococcus pneumoniae* infection induces the production of mitochondrial ROS (mtROS), which can lead to intracellular autophagy via inhibition of mTOR signaling. Other research has shown that swine-origin *Streptococcus* can express superoxide dismutase A, which effectively downregulate the level of intracellular bactericidal autophagy through inhibiting the release of ROS (Fang et al., 2015; Li et al., 2015). LPS is a main component of gram-negative bacteria. Stimulation of macrophages by LPS induced mtROS production and activation of autophagy. LPS-induced NOX2 expression and activated MAPK signaling pathway are involved in the process (Wang et al., 2020). Dengue virus and pseudorabies virus can directly damage mitochondria and cause potential mitochondrial transmembrane loss, further inducing autophagy (Kramer and Enquist, 2012; Chatel-Chaix et al., 2016).

**FIGURE 1:**
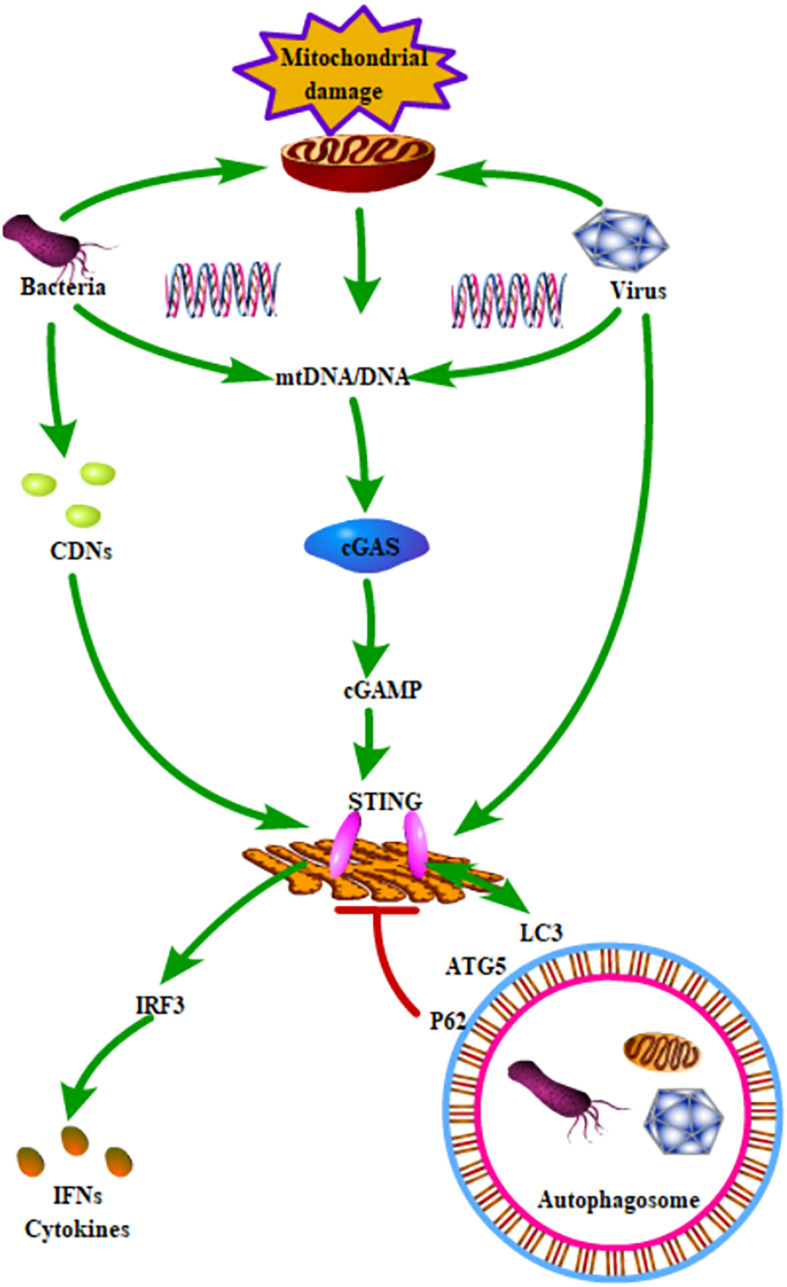
Schematic role of STING signaling in autophagy and mitochondrial homeostasis. STING could be activated by all kinds of pathogenic microorganisms and mtDNA. The activated STING could induce the production of IFNs and cytokines through phosphorylation of IRF3. In addition, STING directly interacted with LC3 and further activated ATG5-dependent autophagy. Moreover, P62, which is a key receptor for regulating autophagy, could degrade STING.

Identifying the signal transduction pathways shared by both autophagy and mitochondrial homeostasis during infection can help us understand the relationship between autophagy and mitochondrial homeostasis. The STING pathway can be activated in two ways. Bacterial CDN s can bind to the STING dimer, activating downstream signaling pathways. Additionally, cytoplasmic dsDNA can be recognized by cGAS, inducing the synthesis of cGAMP, which can bind to STING in the endoplasmic reticulum (Burdette et al., 2011). CGAS can recognize a variety of DNA from multiple sources, including cytosolic pathogen-derived DNA or self-DNA. When the mitochondrial redox homeostasis is out of balance, mtDNA is released into the cytosol, where it can be recognized by cGAS, further promoting STING-mediated signaling pathways (West et al., 2015). Studies generally thought that STING activation is mainly involved in inflammation and type I IFN response. However, a recent study found that STING directly activates autophagy during HSV-1 infection (Liu et al., 2019). Activated STING can directly interact with LC3 via its LC-3 interacting regions and induce the non-canonical pathway of autophagy (Figure 1). It is generally thought that autophagy helps to maintain cellular homeostasis by clearing intracellular harmful substances.

**FIGURE 2:**
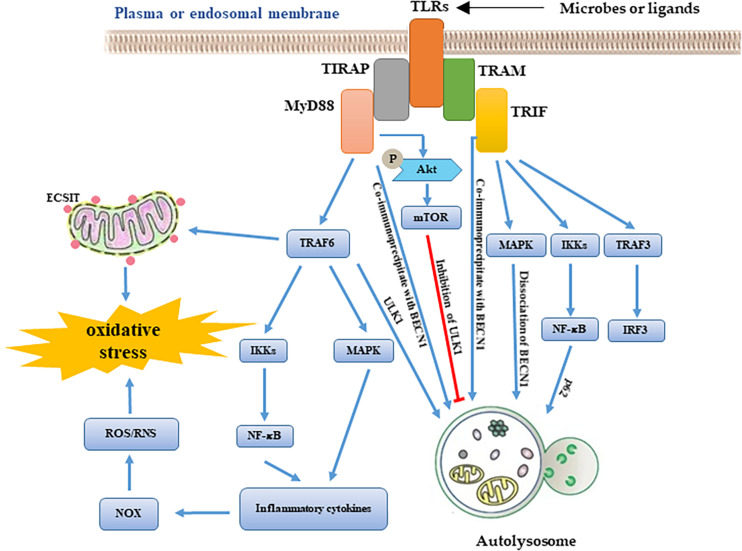
Schematic roles of TLRs signaling in autophagy and mitochondrial homeostasis. According to the availability of adaptor molecules, activation of TLRs signaling induces MyD88-TRAF6 pathway and TRIF pathway. On one hand, TLRs downstream signaling molecules affect mitochondrial homeostasis via regulating ROS production. On another hand, these molecules are also involved in the formation of autolysosome through regulates activity of autophagy-related genes.

It is well-known that TLRs are important PRRs. The activation of TLR1/2/4 leads to the recruitment of mitochondria to the phagosome and induces the production of mtROS. TLR-mediated activation of TRAF6 is a key event that leads to ECSIT ubiquitination around mitochondria, which promotes increasing the mtROS level (Vogel et al., 2007; West et al., 2011). In addition, several studies also show the links between mitochondria and TLR signaling. LPS stimulation increases the production of ROS through translocating NFAT1 into mitochondria (Ma et al., 2015). TLR2 and TLR4 are involved in the induction of mitochondrial biogenesis during *S. aureus* infection (Sweeney et al., 2010). Further research has shown that the inhibition of mtROS promotes *Salmonella* infection (West et al., 2011). There are also studies which show that suppression of ROS release helps to clear pathogens via redox signaling (Paiva and Bozza, 2014). Thus, we hypothesized that the dual-role of autophagy might lead to these opposite results. Autophagy is thought to be an effector of TLR signaling (Delgado et al., 2009). LPS-induced autophagy depends on the TLR4-MyD88-p38 MAPK pathway (Xu et al., 2007; Wang et al., 2020). SsRNA-induced autophagy depends on TLR7-MyD88-mediated regulation of Beclin-1, ATG5, and p62 expression (Delgado et al., 2008; Li et al., 2016). RNA virus-induced autophagy depends on TLR3-TRIF pathway (Gao et al., 2018; de Carvalho et al., 2019). TLR signaling is involved in the regulation of the MyD88, TRIF, MAPK, and PI3K pathways. MyD88 and TRIF can co-immunoprecipitate with Beclin 1, which reduces the binding of Beclin 1 to Bcl-2, inducing autophagy (Shi and Kehrl, 2008). MAPK signaling affects the maturation step of autophagy via mTOR signaling (Zhou et al., 2018). TLRs can affect Akt phosphorylation which regulates the formation of autophagosome via PI3K and mTOR signaling (Shariq et al., 2021). These studies suggest that TLR signaling is an important bridge between autophagy and mitochondrial function during infection (Figure 2).

AMPK is an evolutionarily conserved serine/threonine protein kinase that can be activated by all kinds of physiological or pathological stimulation. AMPK is also an essential initial signal of autophagy. AMPK-induced autophagy is mainly concerned with the inhibition of mTOR and phosphorylation of the ULK1 complex (Li and Chen, 2019). AMPK mediated mTORC1 inactivation through the phosphorylation of the tuberous sclerosis complex, which transforms the active RHEB-GTP into inactive RHEB-GDP. In addition, AMPK can directly interact with the Ser/Pro rich region of ULK1 and induce ULK1 phosphorylation. Subsequently, activated ULK1 promotes its own interaction with ATG13, ATG101, and FIP200 and increases the activity of the ULK1 complex, which is involved in the biogenesis of autophagosomes (Tamargo-Gomez and Marino, 2018). Moreover, AMPK also can directly phosphorylate FOXO3 and further induce the translocation of ATG under conditions of stress (Tamargo-Gomez and Marino, 2018). AMPK is also involved in affecting mitochondrial biogenesis and dynamics. There is direct evidence that dominant-negative mutants of AMPK cannot induce mitochondrial biogenesis in mice (Zong et al., 2002). Further research has suggested that AMPK directly phosphorylates PGC1α, a major regulator of the mitochondrial biogenesis, at Thr177 and Ser538 (Lin et al., 2002). In addition, AMPK can indirectly activate PGC1α via the p38 MAPK and TFEB signaling pathway (Settembre et al., 2013; Wu et al., 2015). Furthermore, AMPK is involved in ER stress, which can trigger autophagy via IRE1α and PERK signaling (Kouroku et al., 2007). Activation of AMPK by its specific small-molecule activator can trigger mitochondrial fission without mitochondrial damage (Toyama et al., 2016). It has been found that mitochondrial fission factor (MFF) could be phosphorylated by AMPK (Ducommun et al., 2015). Phosphorylation of MFF triggers mitochondrial fragmentation through regulating dynamin-like protein 1 (Loson et al., 2013). These interesting studies reveal how AMPK signaling links autophagy and mitochondrial function.

## Conclusion and Perspectives

Autophagy has dual-roles during pathogenic infection—facilitating the clearance of pathogens and promoting the survival of pathogens. The ability of mitochondria to function normally during infection is an important factor that influences autophagy. An optimal amount of mitochondrial metabolites or respiratory burst helps to eliminate pathogen. However, too high or too low levels of mitochondrial metabolites promote pathogen survival and further trigger cell death. In addition, autophagy is influenced by various factors, including the type of pathogen, intensity and duration of infection, and type of host cell. However, it is still controversial if the imbalance of mitochondrial homeostasis induces autophagy or if autophagy helps to maintain mitochondrial homeostasis. For example, GPX4 usually helps to maintain intracellular redox equilibrium and protects intracellular membrane structures against lipid peroxidation (Yang et al., 2014). However, a recent study found that STING is carbonylated at C88 via lipid peroxidation in a GPX4 mutant, which inhibits STING (Jia et al., 2020). The GPX4 mutant also had excessive lipid peroxidation of mitochondria, which promoted the release of mtDNA into the cytoplasm (Pessayre et al., 2004; Tadokoro et al., 2020). The cytoplasmic mtDNA induced formation of cGAMP and further activated STING. However, the specific mechanism regarding these two processes requires further research.

Understanding the mechanisms by which homeostasis is maintained in organisms is one of the most important steps in combatting infection. In this review, we first list the functions of autophagy and mitochondria during infection. Numerous studies have shown that autophagy and the normal functions of mitochondria are central elements in controlling pathogenic infection. However, a variety of pathogens can utilize autophagy and mitochondria to achieve their own survival and proliferation. Moreover, we summarized several links between mitochondrial homeostasis and autophagy, including cGAS-STING signaling, TLR signaling, and AMPK signaling—all of which are involved in the recognition of pathogens, initiation and induction of autophagy, and mitochondrial biogenesis and dynamics. These signaling pathways should be targets for future antiviral and antibacterial studies.

## Author Contributions

SW and KZ conceptualized and wrote this manuscript. YY assisted with the edited version. JL acquired the funding. All authors contributed to the article and approved the submitted version.

## Conflict of Interest

The authors declare that the research was conducted in the absence of any commercial or financial relationships that could be construed as a potential conflict of interest.

## Publisher’s Note

All claims expressed in this article are solely those of the authors and do not necessarily represent those of their affiliated organizations, or those of the publisher, the editors and the reviewers. Any product that may be evaluated in this article, or claim that may be made by its manufacturer, is not guaranteed or endorsed by the publisher.

## Abbreviations

AMPK: AMP-activated Kinase
ARHGDIA: Rho GDP Dissociation Inhibitor Alpha
ATG: Autophagy related gen or protein
Bcl-2: B cell lymphoma 2
Beclin-1: Coiled-Coil Moesin-Like BCL2-Interacting Protein
CDN: Cyclic dinucleotide
cGAMP: Cyclic GMP-AMP
cGAS: Cyclic GMP-AMP Synthase
ECSIT: Evolutionarily Conserved Signaling Intermediate in Toll Pathway
FIP200: Focal adhesion kinase family interacting protein of 200 kD
GPX4: Glutathione Peroxidase 4
LC3: Microtubule-associated proteins 1A/1B light chain 3B
MAPK: Mitogen-Activated Protein Kinase
MEF: Mitochondrial fission factor
MHC: Major histocompatibility complex
mtDNA: Mitochondrial DNA
MOMP: Mitochondrial membrane permeabilization
mTOR: Mammalian target of rapamycin
MyD88: Myeloid Differentiation Primary Response 88
NFAT1: Nuclear factor of activated T-cells 1
NLRP3: NLR Family Pyrin Domain Containing 3
NLRs: Nod-like receptors
NO: Nitric oxide
NOX: Nitrogen oxide
OPTN: Optineurin
OxyR: A member of LysR family of transcriptional regulation
p62: Sequestosome 1, also known as SQSTM1
PAMPs: Pathogen-associated molecular patterns
PERK: Proline-rich receptor-like protein kinase
PI3K: Phosphatidylinositol 3 kinase
PRRs: Pattern recognition receptor
RNS: Reactive nitrogen species
ROS: Reactive oxygen species
SCVs: Salmonella-containing vacuoles
SHISA-5: Shisa Family Member 5
STING: Stimulator of interferon genes
T4SS: Type IV secretion system
TFEB: Transcription Factor EB
TLRs: Toll-like receptors
TRAF6: Tumor necrosis factor receptor (TNFR)-associated factor 6
TRIF: Toll/IL-1R domain-containing adaptor-inducing IFN-beta
ULK1: Unc-51-like kinase 1.
